# Occurrence and distribution of extended-spectrum β-lactamase in clinical *Escherichia coli* isolates at Ho Teaching Hospital in Ghana

**DOI:** 10.4314/gmj.v55i4.11

**Published:** 2021-12

**Authors:** John G Deku, Kwabena O Duedu, Emmanuel Ativi, Godsway E Kpene, Patrick K Feglo

**Affiliations:** 1 Department of Medical Laboratory Sciences, School of Allied Health Sciences, University of Health and Allied Sciences, Ho, Ghana; 2 Department of Biomedical Science, School of Basic and Biomedical Sciences, University of Health and Allied Sciences, Ho, Ghana; 3 Department of Clinical Microbiology, School of Medicine and Dentistry, KNUST, Kumasi, Ghana

**Keywords:** *Escherichia coli*, Extended Spectrum β-Lactamase, Combination disc method, Phenotypic detection, Polymerase Chain Reaction

## Abstract

**Objective:**

This study determined the occurrence and distribution of Extended Spectrum β-Lactamase (ESBL) genotypes of *E. coli* isolates in Ho Teaching Hospital, Ghana.

**Design:**

A cross-sectional study.

**Setting:**

A single centre study was conducted at Ho Teaching Hospital of Ghana.

**Participants:**

Patients who visited Ho Teaching Hospital Laboratory with the request for culture and susceptibility testing.

**Main outcome measure:**

*Escherichia coli* were isolated, and Extended-Spectrum β-Lactamase genes were detected.

**Results:**

Of the 135 isolates, 56(41.5%,95% CI: 33.1% – 50.3%) were ESBL producers. More males, 14(58.3%), produced ESBL than females, 42(37.8%). The ESBL prevalence was highest among the elderly who were 80 years and above 3(100.0%), with the least prevalence among patients within 50–59 years and 0–9 years age bracket, representing 4(25.0%) and 3(27.3%), respectively. The total prevalence of ESBL was marginally higher among out-patients (41.8% 95% CI: 31.9% – 52.2%) compared to in-patients [40.5% 95% CI: 24.8% – 57.9]. BlaTEM-1 was the predominant ESBL genotype obtained from 83.9% (47/56) of the confirmed ESBL producing isolates, with the least being TOHO-1 4(7.1%). The co-existence of 2 different ESBL genes occurred in 19(33.9%) of the isolates. The single and quadruple carriage were 16(28.6%) and 3(5.4%), respectively. The highest co-existence of the ESBL genotypes was recorded for blaTEM-1 and blaCTXM-1 15(26.8%), followed by blaTEM-1, blaCTXM-1 and blaSHV-73 [12(21.4%)].

**Conclusion:**

The high prevalence of ESBL-producing *E. coli* isolates with multiple resistant gene carriage is a threat to healthcare in the study area.

**Funding:**

This research received no external funding.

## Introduction

Extended-Spectrum β-Lactamases (ESBLs) are enzymes that confer resistance to β-lactam antibiotics, such as cephalosporins, aztreonam, penicillin and monobactams. The enzymes were described and first detected in *Klebsiella* and later in *Escherichia coli* and other Gramnegative rods. 1,2 There are different ESBL enzyme types, but the commonest one includes Temoneira (TEM β-lactamase), Cefotaximase (CTX-M β-lactamase) and Sulfhydryl variable (SHV β-lactamase).^3^ These ESBLs may have emerged due to the previous usage of 3rd and 4th generation cephalosporins, recent hospitalisation, recent use of antibiotics, patients over 60 years of age, diabetes, among others.^4^,^5^ The enzymes have substrate specificity and an affinity to the cephalosporin types introduced into hospital use. These enzymes are plasmidborne and are spread from one organism to another by conjugation. The enzymes also spread from person to person and in hospital wards from where they spread to communities. This ESBL producing *E. coli* is a threat to public health as it has been associated with increasing morbidity and mortality.^6^ In a study in Indonesia, Sianipar *et al.* 7 reported a mortality rate of 30.6% in patients infected with ESBL-producing bacteria.

In the United Kingdom, the mortality rate from bacteraemia infection caused by ESBL-producing *E. coli* was 60.9% as against 27.3% from those that did not produce ESBL. 8 In their systematic review and meta-analysis in Ethiopia, Tufa *et al.* 9 reported a death rate of 33.3% and 100.0% due to infections caused by ESBL-producing gram-negative isolates in two separate studies. Infection with ESBL-producing organisms is severe in developing countries like Ghana. There is a scarcity of information on the subject, coupled with misuse of drugs and high antibiotic resistance rates.^10^

Some studies have reported the prevalence of ESBL producing *E. coli*, especially among patients with urinary tract and bloodstream infections. A study in China undertaken by Quan et al. 11 reported an ESBL prevalence of 55.5% in *E. coli* isolated from patients with communityonset bloodstream infections. In their study at Bhubaneswar in India, Singh and other co-researchers^12^ also reported 61.1% ESBL prevalence in *E. coli* isolates among patients admitted to the intensive care unit of a tertiary hospital. Varying levels of ESBL production were recorded in the Potohar region of Pakistan (40.0%) 13 and other countries across the globe. 7,14–16 In Africa, Hertz et al. in Nigeria reported a prevalence of 53.3% in their study to determine ESBL production in *E. coli* organisms.^17^ Similar studies carried out in other Africa countries reported 35.5% prevalence in Cotonou, Benin 18, 22.3% in Malawi 19, 6.7% in North-Western Libya 20 and 59.7% in Egypt 21, among others. In Komfo Anokye Teaching Hospital in Kumasi, Ghana, Feglo, Adu-Sarkodie^22^ recorded a prevalence of 49.4% in *E. coli* isolates they studied. Comparatively, a higher prevalence of 84.3% was reported in Brong-Ahafo Regional Hospital in Ghana by Wireko *et al.* 23

The emergence and dissemination of ESBLs in *E. coli* isolates in health care settings are of great concern, as they lead to treatment failures with cephalosporins. 6,10 Generally, there are limited comprehensive data on ESBL-producing *Enterobacteriaceae* in African countries since most health facilities cannot test for the presence of these enzymes. This study aimed at determining *E. coli* ESBL occurrence and genotypes circulating in Ho Teaching Hospital (HTH) of Ghana.

## Methods

### Study area and design

A cross-sectional study design was used for the study. It was done at the HTH and the School of Basic and Biomedical Sciences research laboratory, the University of Health and Allied Sciences, Ho, over 24 months (20^th^ April 2018–19^th^ April 2020). The HTH is a 241-bed capacity tertiary medical facility located in the capital of the Volta Region, Ho.

The microbiology department of the hospital receives a request for various microbiological analyses from various units and departments of the hospital, including out patient's department, male ward, female ward, paediatric ward, accident and emergency unit, dental unit, dialysis unit, and ear, nose and throat units, among others.

### The bacterial isolates

*E. coli* isolates were cultured from various clinical specimens, including urine, high vaginal swabs and blood on MacConkey agar and blood agar. Growths suspected of *E. coli* were confirmed using Gram stain reaction, triple sugar fermentation test, citrate test, urease test, indole test, Voges Proskauer and methyl red test. Organisms isolated and confirmed as *E. coli* were inoculated into 80% glycerol-Mueller Hinton broth, stored in a -80°C freezer, and later used for other tests. Control organisms were *Escherichia coli* (ATCC 25922) and *Klebsiella pneumoniae* (NCTC 13442).

### Phenotypic detection of ESBL

#### Screening of *E. coli* isolates for ESBL production

Screening *E. coli* for ESBL production was done by subjecting the isolates to cefpodoxime (10µg), ceftazidime (30µg) and ceftriaxone (30µg), according to the procedures described by CLSI 2018. 24After removing the stored isolates from the freezer, the surface was aseptically scraped and subcultured onto Nutrient agar. After overnight incubation, the *E. coli* isolates from Nutrient agar were emulsified in buffered phosphate saline. The turbidity was measured with a DensiCHEK plus densitometer (Biomerieux, U.S.) to obtain 0.5 McFarland standards. The isolates exhibited a zone of inhibition of ≤17mm, ≤22mm or ≤25mm to at least one of the three antimicrobial agents; cefpodoxime, ceftazidime or ceftriaxone, respectively, as screen positive for ESBL production. The positive screen isolates were subjected to ESBL confirmation by the combination disc method.

### Confirmation of ESBL production by combination disc method

Screen positive *E. coli* isolates were confirmed for ESBL production using the combination disc (Oxoid, UK) method. After removing the stored isolates1 from the freezer, the surface was aseptically scraped and subcultured onto Nutrient agar. After overnight incubation, *E. coli* isolates from Nutrient agar were emulsified in buffered phosphate saline, and the turbidity was adjusted to 0.5 McFarland standards. The culture was used to seed the Muller-Hinton agar surface evenly. The antibiotic discs were then applied. After overnight incubation, the zones of inhibition around the cefpodoxime disc alone and that of cefpodoxime plus clavulanic acid were measured and recorded. ESBL production was detected when the difference between the zone of inhibition around the cefpodoxime alone and around cefpodoxime/clavulanic acid was 5mm or greater and interpreted according to the procedure of Clinical and Laboratory Standards Institute (CLSI). 24

### Molecular detection of ESBL

#### DNA Extraction

An overnight culture of the isolates in 30ml Luria Bertani broth (Oxoid, UK) in a shaking incubator was used for the genomic DNA extraction using high molecular weight phenol-chloroform extraction method 25, except that Tris EDTA (TE) was used as the elution buffer. The harvested DNA was incubated at 4°C for two days to resuspend the pellet into a translucent viscous gel. The concentration of the extracted DNA was measured using a Nanodrop spectrophotometer (Thermo Scientific), and the viscous DNA was stored under -24°C.

### Detection of ESBL genes by Polymerase Chain Reaction

*E. coli* ESBL genotypes were determined using Polymerase Chain Reaction (PCR). ESBL genotypes tested for include *Bla*TEM-1, B*bla*CTXM-1, *Bla*CTXM-825, *Bla*CTXM-914, *Bla*SHV-73 and TOHO-1. The primer sequence and their annealing temperatures are listed in Table 1. The PCR conditions were: initial denaturation at 94°C for 30 seconds, actual denaturation at 94°C for 30 seconds, 45 seconds of annealing at 62°C for *Bla*CTXM-914, and 55°C for *Bla*CTXM-1, *Bla*CTXM-825, *Bla*TEM-1, *Bla*SHV-73 and TOHO-1. Initial and final extensions were done at 68°C for 60 seconds per kilobase and 5 minutes, respectively. PCR reaction volume was 12.5µl for each primer pair and the components of the reaction were 6.25µl of one taq quick-load 2X master mix with standard buffer, 0.25µl each of forward and reverse primers and 4.75µl of nuclease-free water. Thermocycling was done for 30 cycles.

**Table 1:** Oligonucleotide primers used for the detection of ESBL genes

Target gene	Sequence (5′ - 3′)	Product size (bp)	Reference
*Bla*CTXM-1	F_GAC GAT GTC ACT GGC TGA GC	499	26
	R_ AGC CG C CGA CGC TAA TAC A		
*Bla*CTXM-825	F_CGC TTT GCC ATG TGC AGC ACC	307	26
	R_GCT CAG TAC GAT CGA GCC		
*Bla*CTXM-914	F_GCT GGA GAA AAG CAG CGG AG	474	26
	R_GTA AGC TGA CGC AAC GTC TG		
TOHO 1	F_GCG ACC TGG TTA ACT ACA ATC C	351	26
	R_CGG TAG TAT TGC CCT TAA GCC		
*Bla*TEM-1	F_CAACATTTTCGTGTCGCCCTT	496	27
	R_TTCATTCAGCTCCGGTTCCCA		
*Bla*SHV-73	F_CGCTTTCCCATGATGAGCACCT	320	27
	R_CGCCTCATTCAGTTCCGTTTCCC		

### Loading of amplicons

Using a micropipette, the resulting PCR product was loaded into agarose wells. The first and second lanes were loaded with 6µl of 100bp ladder and 10µl of a known DNA positive for CTXM, SHV, TEM or TOHO. The last lane was loaded with a known negative sample. The remaining lanes were loaded with samples under investigation.

### Visualisation of amplicons

**Figure 1:**
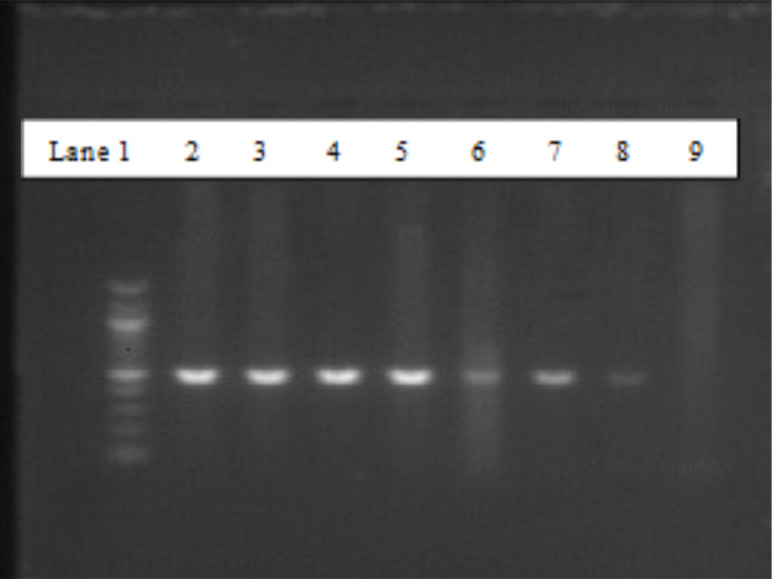
PCR gel image of *Bla*CTXM-1. Lane 1 was 100bp. DNA ladder. Lanes 2 and 9 represented positive and negative controls, respectively. Lanes 3-8 were samples tested for *Bla*CTXM-1

After the electrophoresis, the resulting gel was visualised using an image analysis system (UVITEC Cambridge, United Kingdom). The images were photographed with a digital camera (Kodak, Japan), as shown in Figure 1, to detect the BlaCTXM-1 gene.

### Ethical approval

Permission to carry out this study was granted by the management of the Ho Teaching Hospital (Ref No.: VRH/1/102). Ethical clearance for the study was granted by the Joint Committee on Human Research, Publication and Ethics, School of Medical Sciences and Komfo Anokye Teaching Hospital, with the protocol number CHRPE/AP/204/18.

### Statistical analysis

All statistical analyses were performed using IBM-SPSS Statistics for Windows, version 26 (IBM Corp., Armonk, N.Y., USA), Microsoft Excel 2016 and GraphPad Prism 8.0 (GraphPad Software, Inc., San Diego, CA). Descriptive outcomes for a categorical variable were presented as counts and percentages in tables and figures. Bivariate analysis was performed using the Pearson chi-square test for categorical variables and Fisher's Exact Test, where there were only two categorical outcomes in the independent variable. Chi-square test for trend was also computed to test the association between age as an ordinal variable and ESBL production. Two-sided P-values <0.05 were considered as significant results.

## Results

This study involved 135 clinical isolates of *E. coli* from various human clinical specimens. Of the 135 clinical isolates collected, the majority of 111(82.2%) were from females. Christianity was identified as the most professed religion among the patients constituting 94.8% of the study participants. The majority of the patients, 33(24.4%), were within the age brackets of 30 and 39 years. The least represented age group was patients between 10–19 years, accounting for 1.48%. While a considerable proportion of 72.6% of the patients were general out-patient attendants, a few were in-patients. Most of the *E. coli* isolates, 98(72.6%), were obtained from the urine specimens, with pleural aspirate being the least 1(0.7%) (Table 2).

**Table 2:** Sociodemographic and clinical characteristics of study population

Parameter	Frequency (%)
**Gender**	
**Male**	24(17.8)
**Female**	111(82.2)
**Age group**	
**0 – 9**	11(8.2)
**10 – 19**	2(1.5)
**20 – 29**	25(18.5)
**30 – 39**	33(24.4)
**40 – 49**	19(14.1)
**50 – 59**	16(11.9)
**60 – 69**	17(12.6)
**70 – 79**	9(6.7)
**80 years and above**	3(2.2)
**Religion**	
**Christianity**	128(94.8)
**Islam**	2(1.5)
**None**	5(3.7)
**Marital Status**	
**Single**	46(34.1)
**Married**	84(62.2)
**Cohabitation**	3(2.2)
**Widowed**	2(1.5)
**Specimen Type**	
**Urine**	98(72.6)
**Wound**	14(10.4)
**High vaginal swab**	10(7.4)
**Blood**	5(3.7)
**Ear swab**	5(3.7)
**Sputum**	2(1.5)
**Pleural aspirate**	1(0.7)
**Patient Status**	
**Out-Patient**	98(72.6)
**In-Patient**	37(27.4)
**Wards**	
**A/E**	1(0.7)
**F2**	1(0.7)
**G1**	4(3.0)
**G2**	3(2.2)
**G3**	3(2.2)
**G4**	7(5.2)
**ART**	18(13.3)
**Total**	**135(100.0)**

Data presented as count and percentages.

OPD: Outpatients department, A/E: Accident and emergency, F2: Children's ward, G1: Urology unit, G2: Female surgical, G3: Male surgical, G4: Gynaecological ward, ART: Anti-retroviral therapy unit.

A total of 56 out of the 135 isolates were confirmed to have ESBL, representing a prevalence of 41.5% (95% CI: 33.1% – 50.3%). Although statistically not significant, the burden of ESBL positivity was higher in isolates obtained from males than females. The preponderance of ESBL positivity was highest among the elderly [≥80 years 3(100.0%)] with the least occurrence in patients within 50–59 years (4) and 0–9 years age (3) bracket, representing 25.0% and 27.3% respectively (Table 3).

**Table 3:** Prevalence and risk of association of ESBL among *E. coli* clinical isolates

Parameter	Negative (%)	Positive (%)	P-value
**Gender**			
Male	10(41.7)	14(58.3)	0.072
Female	69(62.2)	42(37.8)	
**Age group** **(years)**			
0 – 9	8(72.7)	3(27.3)	0.313
10 – 19	1(50.0)	1(50.0)	
20 – 29	17(68.0)	8(32.0)	
30 – 39	18(54.6)	15(45.5)	
40 – 49	9(47.4)	10(52.6)	
50 – 59	12(75.0)	4(25.0)	
60 – 69	9(52.9)	8(47.1)	
70 – 79	5(55.6)	4(44.4)	
80 and above	0(0.0)	3(100.0)	
**Marital status**			
Single	30(65.2)	16(34.8)	0.274
Married	45(53.6)	39(46.4)	
Co-habitation	3(100.0)	0(0.0)	
Widowed	1(50.0)	1(50.0)	
**Religion**			
Christianity	75(58.6)	53(41.4)	0.152
Islam	0(0.0)	2(100.0)	
None	4(80.0)	1(20.0)	
**Patient status**			
Out-patient	57(58.2)	41(41.8)	1.000
In-patient	22(59.5)	15(40.5)	
**Total**	**79(58.5)**	**56(41.5)**	

This study showed that all 1(100.00%) of the *E. coli* isolated from pleural aspirate was confirmed to contain ESBL. 64.3% (95% CI: 35.1% – 87.2%) of the isolates obtained from the wound were ESBL producers.

Less than half (<50%) of the isolates obtained from blood, ear swab, high vaginal swab and urine were confirmed to be carrying ESBL genes. Details of these results are presented in Table 4.

**Table 4:** Distribution of ESBL based on the anatomical source of the specimen from which the *E. coli* were isolated

Specimen Type	Total	ESBL (Positive)	Proportion (%)	95% CI
**Urine**	98	38	38.8	29.1 – 49.2
**Wound**	14	9	64.3	35.2 – 87.2
**HVS**	10	3	30.0	6.7 – 65.2
**Blood**	5	2	40.0	5.3 – 85.3
**Ear**	5	2	40.0	5.3 – 85.3
**Sputum**	2	1	50.0	5.3 – 85.3
**Pleural Aspirate**	1	1	100.0	-

**Figure 2:**
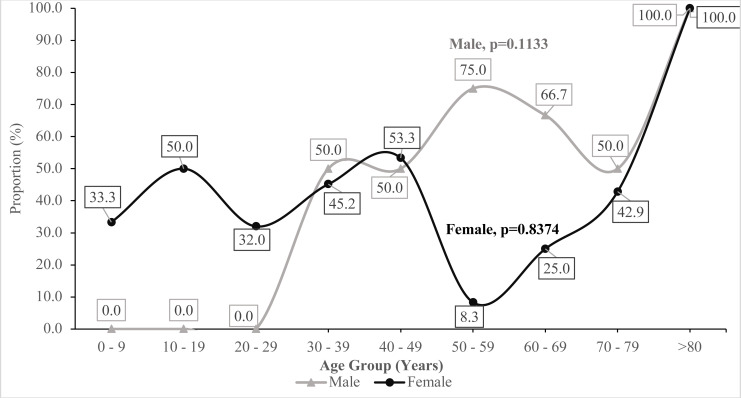
Trend of ESBL occurrence across increasing age categories stratified by gender of the patients

Generally, there was an increasing undulating trend of ESBL across the increasing age categories for males from the least burden of 0.0% among the age group 0–9 years through to 100.0% among those aged 80 years and above. A similar pattern of rising and fall ESBL-positivity was observed across the female age groups with a trough at age 50 – 59 years (8.3%) and crest at age group 80 years and above (Figure 2).

**Figure 3:**
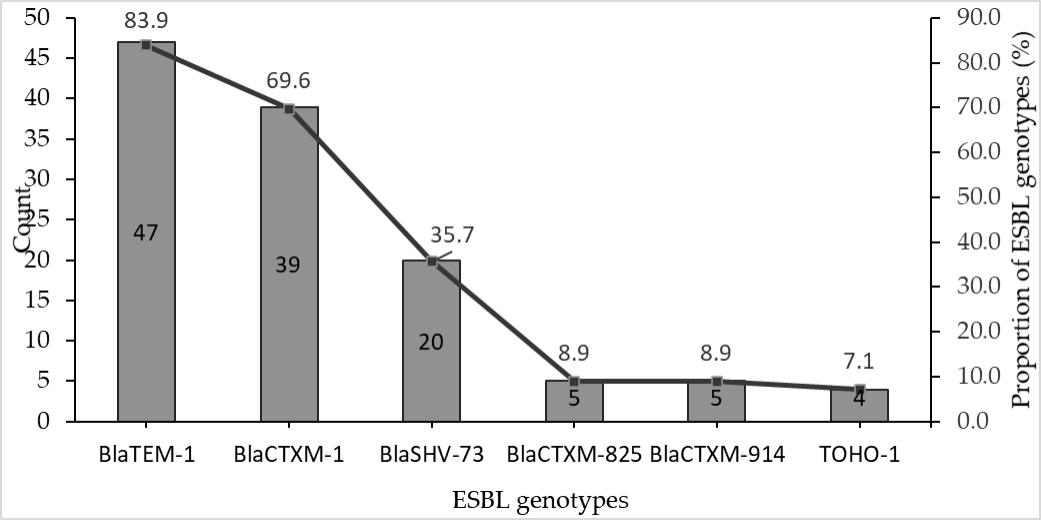
Occurrence of ESBL genotypes in *E. coli* isolates in Ho Teaching Hospital.

*Bla*TEM-1 was the predominant ESBL genotype and was detected in 83.9% (47/56) of the *E. coli* isolates. *Bla*CTXM-1 and BlaSHV-73 were seen in 69.6% and 35.7% of the confirmed ESBL producing isolates. Less than 10% of the ESBL positive isolates contained *Bla*CTXM-825, *Bla*CTXM-914 and TOHO-1 genes accounting for 8.9%, 8.9% and 7.1%, respectively (Figure 3).

The highest burden of ESBL genotypes was found among those between 30 and 39 years, and 40 and 49 years except *Bla*CTXM-914, which was not detected in the isolates obtained from these age brackets. The lowest frequency of the ESBL genes was obtained from patients between 10 and 19 years. Except for the ESBL genotype of the *Bla*CTXM-1 gene, where proportions were higher among the females than the males, the male gender had higher percentages of all the other five ESBL genes in this study. Similarly, inpatients had a greater percentage of the ESBL genotypes except for *Bla*CTXM-825, even though the frequency of the carriage was higher in the out-patients. The total ESBL prevalence was, however, higher in out-patients 41(41.8%) than among the in-patients 15(40.5%) (Table 5).

**Table 5:** Sociodemographic characteristics and ESBL gene distribution

Parameter	Count (%)	blaTEM-1	blaSHV-73	blaCTXM-1	blaCTXM-825	blaCTXM-914	TOHO
**Gender**							
Male	14(58.3)	13(92.9)	6(42.9)	9(64.3)	2(14.3)	2(14.3)	2(14.3)
Female	42(37.8)	34(81.0)	14(33.3)	30(71.4)	3(7.1)	3(7.1)	2(4.8)
**Patient status**							
Out-patient	41(41.8)	34(82.9)	14(34.2)	28(68.3)	5(12.2)	3(7.3)	2(4.9)
In-patient	15(40.5)	13(86.7)	6(40.0)	11(73.3)	0(0.0)	2(13.3)	2(13.3)
**Age group**							
0–9	3(27.3)	3(100.0)	0(0.0)	2(66.7)	1(33.3)	0(0.0)	0(0.0)
10–19	1(50.0)	1(100.0)	1(100.0)	1(100.0)	0(0.0)	0(0.0)	0(0.0)
20–29	8(32.0)	7(87.5)	3(37.5)	6(75.0)	0(0.0)	0(0.0)	0(0.0)
30–39	15(45.5)	13(86.7)	7(46.7)	9(60.0)	2(13.3)	0(0.0)	1(6.7)
40–49	10(52.6)	6(60.0)	3(30.0)	7(70.0)	0(0.0)	0(0.0)	0(0.0)
50–59	4(25.0)	4(100.0)	0(0.0)	2(50.0)	0(0.0)	0(0.0)	0(0.0)
60–69	8(47.1)	7(87.5)	4(50.0)	6(75.0)	2(25.0)	2(25.0)	2(25.0)
70–79	4(44.4)	4(100.0)	2(50.0)	3(75.0)	0(0.0)	1(25.0)	0(0.0)
≥80	3(100.0)	2(66.7)	0(0.0)	3(100.0)	0(0.0	0(0.0)	0(0.00)

0.00=not detected

The ESBL genes *Bla*SHV-73, *Bla*CTXM-825, *Bla*CTXM-914, and TOHO-1 were predominantly seen in non-urine samples than in urine samples. All the 6 ESBL genotypes under investigation were found in the isolates obtained from wound and urine. *Bla*CTX-M-1 was the only genotype carried by the isolates obtained from pleural aspirate (Table 6).

**Table 6:** Distribution of ESBL genes based on the anatomical source of the specimen

	Total count (%)	*Bla*TEM-1	blaSHV-73	blaCTXM-1	blaCTXM-825	blaCTXM-914	TOHO-1
**Specimen type**							
Blood	2(40.0)	2(100.0)	0(0.0)	1(50.0)	0(0.0)	0(0.0)	0(0.0)
Ear swab	2(40.0)	1(50.0)	0(0.0)	1(50.0)	0(0.0)	0(0.0)	0(0.0)
HVS	3(30.0)	3(100.0)	1(33.3)	3(100.0)	1(33.3)	0(0.0)	0(0.0)
Pleural aspirate	1(100.0)	0(0.0)	0(0.0)	1(100.0)	0(0.0)	0(0.0)	0(0.0)
Sputum	1(50.0)	1(100.0)	0(0.0)	1(100.0)	0(0.0)	0(0.0)	0(0.0)
Wound	9(64.3)	8(88.9)	6(66.7)	5(55.6)	2(22.2)	2(22.2)	2(22.2)
Urine	38(38.8)	32(84.2)	13(34.2)	27(71.1)	2(5.3)	3(7.9)	2(5.3)

0.0 = not detected

It was found that 16 of the ESBL producing isolates had one ESBL encoding gene. However, most isolates (19) carried two different ESBL genes, with 3 isolates having four different ESBL genes. Fifteen of the 56 *E. coli* isolates carried BlaTEM-1 and BlaCTXM-1 genes. Details of these results are presented in Table 7.

**Table 7:** Frequency of carriage of the ESBL genes

Resistant gene	Number of isolates carrying the resistant gene (%)
**Single gene carriage**	
**BlaTEM-1**	8 (14.3)
**BlaCTXM-1**	4 (7.1)
**BlaSHV-73**	3 (5.4)
**TOHO-1**	1 (1.8)
**BlaCTXM-914**	0 (0.0)
**BlaCTXM-825**	0 (0.0)
**Total**	**16 (28.6)**
**Double gene carriage**	
**BlaTEM-1 and BlaCTXM-1**	15 (26.8)
**BlaTEM-1 and BlaSHV-73**	2 (3.6)
**BlaTEM-1 and BlaCTXM-914**	1 (1.8)
**BlaCTXM-1 and BlaSHV-73**	1 (1.8)
**Total**	**19 (33.9)**
**Triple gene carriage**	
**BlaTEM-1, BlaSHV-73 and CTXM-1**	12 (21.4)
**BlaTEM-1, CTXM-1 and BlaCTXM-825**	3 (5.4)
**BlaTEM-1, BlaCTXM-914 and TOHO-1**	2 (3.6)
**BlaTEM-1, BlaCTXM-1 and BlaCTXM-914**	1 (1.8)
**Total**	**18 (32.1)**
**Quadruple gene carriage**	
**BlaTEM-1, BlaCTXM-1, BlaCTXM-825** **and BlaSHV-73**	2 (3.6)
**BlaTEM-1, BlaCTXM-1, BlaCTXM-914** **and TOHO-1**	1 (1.8)
**Total**	**3 (5.4)**

## Discussion

Extended-spectrum β-lactamase mediated antimicrobial resistance by *E. coli* isolates has been a global problem. The proportion of *E. coli* developing resistance differs from country to country and even differs within localities in the same country.

The ESBL situation has not been studied at the Ho municipality, but high resistant proportions of *E. coli* were reported from other clinical laboratories. There is, therefore, the need to monitor this occurrence and understand the spread and distribution of ESBL mediated resistance genes in *E. coli*.

In the current study, we recorded an ESBL prevalence of 41.5%. The prevalence reported in this study was lower than those reported in other jurisdictions. A study by Mahamat *et al.* 28 conducted in three different hospitals in Chad reported a prevalence of 63.8%. A similar high burden of ESBL carriage was also recorded in the Brong Ahafo region of Ghana; 84.3% 29, 72.7% in Mali among previously hospitalised bacteraemia patients 30, 92% in South-South Nigeria 31 and 65.2 % in South-Eastern Nigeria.^32^ Other studies recorded low prevalence compared to the finding in this study. 33,34 The burden and distribution of ESBL are dependent on heavy antibiotic use, geographical locations and hospital to hospital variations^35^,^36^, and transmission from community, livestock, animals, and environment 37 and weak infection control measures instituted by various hospitals. 38 Other plausible reasons accounting for this variation in prevalence, according to Abrar *et al.* 39, are the availability of the antibiotics and socio-economic status of the society. This variation in the prevalence may also be due to differences in the type of sample used for analysis and the time of sample collection.^36^ The finding of this study, however, is consistent with the 40.8% prevalence reported by Seyedjavadi *et al.* 36 in their study to determine the relationship among blaTEM, blaSHV and blaCTX-M genotypes in patients suffering from urinary tract infections. The high ESBL prevalence may lead to treatment failure and narrowing the antibiotic spectrum as the enzyme destroys cephalosporins, resulting in therapeutic difficulties. The difficulty in detecting ESBLs and the inconsistencies in reporting ESBLs results have led to a surge in resistance patterns. To curb the spread of antibiotic resistance, there is the need to carry out regular antibiotic usage and resistance surveillance to track the changes in antibiotic susceptibility and formulate appropriate control strategies.

Our study investigated a variety of β-lactamases among the 56 *E. coli* that were phenotypically positive using a combination disc method. These genotypes were *Bla*TEM-1, *Bla*SHV-73, TOHO-1 and *Bla*CTXM (*Bla*CTXM-1, *Bla*CTXM-825 and *Bla*CTXM-914). The predominant ESBL genotype recorded in this study was blaTEM-1 (83.9%), followed by blaCTXM-1(69.6%), with TOHO-1 recording the least prevalence of 7.1%. The high prevalence of BlaTEM-1 was consistent with other reports. In studies performed in Portugal and Turkey, the blaTEM gene was the predominant ESBL gene accounting for 40.9% 40 and 72.7% 41 of ESBL genotypes, respectively. ESBL genotype distribution could be influenced by the type of antibiotics abuse and overuse.

The detection of multiple carriage of two or more betalactam genes within the same organism was reported in this study, where more than half of the ESBL producing *E. coli* isolates carried more than one ESBL gene. This may result in the organism becoming resistant to a wide variety of antimicrobial agents. This study recorded the highest carriage of multiple ESBL genes for blaTEM-1 and blaCTXM-1 genes. The finding of this study corroborates favourably with other findings reported in other countries 36,42. This observation, however, is a threat to public health and will negatively affect treatment outcomes in our healthcare settings. The more the resistance genes on one isolate, the more resistant the organism will be against different antimicrobials that will be used to treat the infection caused by the organism. The carriage of multiple antibiotic resistant genes by an organism has been reported due to the misuse of third-generation cephalosporins.^36^

In this study, patients aged 80 years or more were mostly infected with the ESBL- producing *E. coli*, followed by those within 40–49 years age bracket (52.6%), with the least being 25% for those within 50 and 59 years. No significant relationship existed between the rate of *E. coli* infection and the age of the patients. The outcome of our study is at variance with a report by Seyedjavadi *et al.*^36^, where ageing is a risk factor associated with beta-lactamase mediated resistance in patients infected with *E. coli*. Recovery of ESBL-producing *E. coli* isolates in the aged could be due to the compromised immunity associated with ageing.

Even though not statistically significant, the male gender (58.3%) was mostly infected with the ESBL producing *E. coli* isolates compared to the female counterparts (37.8%). Contrary to our findings, Al-Garni *et al.* 43 reported female preponderance in their study to assess the risk factors and molecular features of extended-spectrum β-lactamase producing bacteria at the southwest of Saudi Arabia, even though the female majority was also not statistically significant. The proportion of women and men with ESBL infections indicates that gender is a non-significant factor for the acquisition of infection.^43^

Despite the findings in this study that high frequency of ESBL producing isolates were obtained from urine samples (38/98) compared to other samples, the prevalence in pleural aspirate was 100.0%. Other studies recorded the most prevalence in blood samples 38 and sputum. 39 The high prevalence of ESBL producing isolates from blood and sputum was contrary to our finding in this study. Most of the ESBL producing *E. coli* isolates were obtained from pleural aspirate and wound swab. Most *E. coli* isolates are commensals in the gut. They can become pathogenic in immunocompromised hosts, causing intestinal and extraintestinal diseases in humans and animals.^44^

### Limitation of the study

The authors acknowledged some limitations in the study. We did not have access to patients' presenting complaints or their initial differential diagnosis. Patients long term history of antibiotic usage were not also known. The study did not investigate the presence of other ESBL genotypes that might be present.

## Conclusion

This occurrence of ESBL-producing *E. coli* isolates in the various samples was high, with blaTEM-1 being the predominant ESBL genotype among the six genotypes identified. Multiple carriage of ESBL genotypes was observed and is of great cause of concern as it may lead to limited therapeutic options. Since antibiotic resistance pattern varies from one region to another, there is also the need to monitor the resistance pattern in other parts of the country.
